# A comparative study of the injury patterns and inflammatory response between suicidal and unintentional falls from height in Germany

**DOI:** 10.1007/s00068-025-03015-1

**Published:** 2025-11-25

**Authors:** Alberto Alfieri Zellner, Marius Robert Schmitt, Jonas Roos, Christian Prangenberg, Henry Pennig, Davide Cucchi, Sebastian Scheidt

**Affiliations:** Department of Orthopaedics and Trauma Surgery, University Hospital Bonn, Venusberg-Campus 1, Bonn, (NRW) 53127 Germany

**Keywords:** Polytrauma, Suicide, Fall, Emergency medicine, Intensive care, Traumatology, Work-related, Fall from height, Inflammatory response, Coagulation

## Abstract

**Background:**

Patients who have fallen from great height with suicidal intent present unique challenges, including delayed medical attention, injury patterns that are often difficult to detect, severe hypothermia, and difficulties obtaining informed consent due to the patient’s mental state. Further delays to treatment can be caused by legal and logistical hurdles, such as coordinating with legal guardians or family members. These co-factors contribute to the high reported overall complication rates in these cases, which can reach up to 50%. This study aims to analyse injury patterns, inflammatory responses and complication rates in people who have jumped to their death, compared to those who have fallen unintentionally. It also aims to investigate the correlation between jump height and injury severity.

**Methods:**

This retrospective monocentric study analysed patient data such as age, gender, injuries, treatments and duration of hospitalisation. A total of 68 suicidal falls were included during the period from January 2014 to January 2024. Subsequently, 68 unintentional falls from a height of more than three meters were included from the same period as a control group, which was analysed alongside the suicidal falls. We analysed injuries sustained in clinical and radiological findings, as well as the inflammatory response and coagulation status of patients in blood gas analysis (BGA) and laboratory analysis of blood drawn in the first hours/days after trauma (C-reactive protein, white blood cell count, lactate, pH value, base excess, procalcitonin and prothrombin time). During hospitalisation, we analysed mortality rates, complication rates, transfusions performed, and intensive care unit/hospital stay for each group.

**Results:**

The group of suicidal jumps had an equal number of male and female patients (50% and 50%, respectively). In contrast, a predominance of male patients was observed in the subgroup of unintentional falls (76.5%, *p* = 0.002). Overall, suicidal falls resulted in a significantly more severe injury pattern. The average mean reported fall height was 9.61 m (± 6.42 m) in the suicidal group compared to 6.14 m (± 4.08 m) in the unintentional group. The injury severity score (ISS) was significantly higher in the suicidal group with 32.04 (± 23.43) compared to 17.37 (± 14.01) in the unintentional group (*p* < 0.001). In accordance with this, higher complication rates (wound healing disorders and total number of complications) were observed in the suicidal group (52.5% vs. 29.9%, *p* = 0.011). A greater number of total surgeries were also required in this group (3.21 ± 4.27 vs. 1.69 ± 2.81, *p* = 0.016). Patients in the suicidal group had significantly worse measurable coagulation status (prothrombin time) upon admission (74.95 (± 21.89) vs. 89.09 (± 21.66), *p* < 0.001), resulting in a significantly higher requirement for transfusions of erythrocyte concentrates (4.07 (± 9.39) vs. 0.97 (± 2.29), *p* < 0.001). Interestingly, comparable trends in ISS, injury patterns, complication rates and coagulation status were observed in a subgroup analysis, even after adjusting the cohorts for fall height (8.41 ± 4.38 m vs. 8.52 ± 3.80 m; *p* = 0.495).

**Conclusion:**

Patients who had attempted suicide by jumping suffered more severe injuries and had a significantly higher ISS than those in the group who had fallen unintentionally. This remained true even when fall height was considered. Suicidal patients exhibited a higher complication rate (*p* = 0.011), increased overall transfusion requirements, impaired coagulation status upon admission and a greater number of operations (including repeat operations) compared to the unintentional fall cohort. Our data suggest that these patients require more resources and are more challenging to treat. We therefore recommend that they be treated in level 1 trauma centres with interdisciplinary intensive care capacities.

## Introduction

In recent years, there has been an increase in reported rates of stress, depression and suicidal behavior across society as a whole [1–3]. For example, Destatis (the Federal Statistical Office of Germany) reported over 9,000 suicides in 2019, roughly three times the number of fatalities on the roads [4]. On a global scale, suicide is a similarly significant issue, with over 800,000 suicides occurring each year. In 2012, it was the second leading cause of death for 15 to 29-year-olds [5–7]. Data collected in the UK and Brisbane City in the years 2008–2012 and 2006–2007, respectively, found that 3–15% of suicides involved jumping from a height [8–10]. This has driven research into trauma surgery and these specific types of injury, with the aim of identifying correlations between trauma mechanisms and injury patterns [11–13]. A specific fracture of the os sacrum has been classified into four subgroups and is known as the “suicide jumper’s fracture” [14, 15]. Another injury that has been clearly observed to be sustained from jumping from a height is a bilateral calcaneus fracture, which was classified by Sanders et al. [16–18]. This injury is relevant and specific to this mechanism of trauma because most falls are suspected to hit with the heels first [19–23]. Many of these high-force blunt traumas, which are often caused by falls from great heights, are classified as polytraumas. They require a high level of care and are therefore primarily treated in Level 1 trauma centers.

According to the Berlin definition (2014), polytrauma is defined as injuries to two or more anatomical regions with an Abbreviated Injury Scale (AIS) score of at least 3, accompanied by at least one concurrent physiological derangement in one of the five specified parameters. The Injury Severity Score (ISS) can be used to assess polytrauma [24, 25]. Thorough examination by paramedics, considering the mechanism of trauma, is important for the preclinical and clinical evaluation and treatment of these patients. Only by identifying these high-energy injuries can the correct diagnostic steps be taken to identify and treat life-threatening conditions correctly, as demonstrated by Scheidt et al. [26, 27]. It is known that high-energy injuries can induce severe inflammatory responses in patients, which can negatively impact wound healing and coagulation status. In some cases, these responses can be used as a prognostic marker for complications and mortality [28–30]. The relationship between severe suicidal fall injuries, the inflammatory response, and subsequent coagulation status has not yet been studied. This research could help us better understand this specific patient group and improve future treatment. Factors specific to this cohort, such as intent, site selection, medication, and intoxication, may affect these patients uniquely.

Self-inflicted harm resulting from suicidal jumps poses additional challenges for treating physicians. Patients who have jumped with suicidal intent may have done so in a location that is difficult for paramedics to reach, or where it takes a significant amount of time for paramedics to be alerted. This is an obvious problem in winter, when patients may suffer severe hypothermia before receiving any medical care [31].

Treatment is further complicated by the fact that informed consent is often unattainable, as suicidal patients are unable to comprehend the risks and consequences of necessary surgical procedures. This means that only critical emergency medical care can be provided. In most cases, a legal guardian is not immediately available, so surgical treatment may be delayed [32]. The treating physician will therefore need to schedule appointments with the legal guardian or the patient’s family members, which would require additional effort. Frequently, it is not possible to obtain a signed informed consent via fax machine, which would be in line with German privacy laws, due to the infrequent use of this technology in other areas of life [33].

These hurdles can negatively impact the outcome for this specific polytrauma group, potentially delaying recovery and increasing complication rates by up to 50% [23, 34].

Our goal is to analyse the specific injury patterns, inflammatory responses and complication rates of suicidal patients in comparison to work-related injuries sustained from falls. Additionally, we aimed to determine whether there is a correlation between jump height and injury patterns.

## Material & methods

A retrospective case analysis was conducted using the hospital’s database between 2014 and 2024. Manual admission tracking was also performed. The inclusion criteria for the suicidal group were falls involving expressed suicidal intent or where suicidal intent was reported by bystanders, and where the patient had jumped in an attempt at self-harm. Suicidal intent was expressed either directly by patients or reported by bystanders/relatives to paramedics, who then conveyed the information to the emergency room team at the Level 1 Trauma Centre. For work- or recreation-related falls, the inclusion criterion was a fall from three meters or above, as this was also the minimum height recorded in suicidal jumps. Manual admission tracking was performed for patients with work- or recreation-related falls until the same number (*n* = 68) as in the suicidal group were included. The time period covered by the study of accidental falls was the same as that covered by the study of suicides: 2014–2024. Injuries identified during physical and radiological examinations were summarized in a table according to the height of the fall, if this information was available. The height was estimated by the paramedics at the scene or calculated based on information about where the fall occurred (one story equals three meters). As most of the suicidal group (*n* = 37, 54.4%) had jumped from a window, and a further 11 patients (16.2%) had jumped from bridges of known height, there was minimal reliance on the estimation of jump height by paramedics. A specialist in radiology and traumatology assessed the radiological exams in each case. The injuries were then further classified according to the Injury Severity Score (ISS), as used in the German polytrauma guidelines [24, 34]. The items used in this study to calculate the ISS were the head, neck, face, chest, abdomen, pelvis and extremities, as well as external injuries. Each item was scored from 0 (no injury) to 6 (fatal). A subgroup analysis was conducted to eliminate the inherent bias resulting from significant differences in fall height between the two groups. For this analysis, the maximum fall height was limited to 25 m for the suicidal group and raised to 5 m for the unintentional group. With these additional inclusion criteria, the suicidal group consisted of 65 patients and the unintentional group of 37.

Due to the limited information provided by some patients and/or emergency physicians, clear suicidal intent was not always documented. Patients for whom clear evidence of suicidal intent was lacking were not included in the suicidal group, but rather assigned to the unintentional group. There were no age restrictions. Furthermore, the patient’s inflammatory response in the form of C-reactive protein, white blood cell count, lactate and base excess were analysed. If available, albumin and vitamin D levels were noted upon arrival at the hospital. Lastly, the time of admission was documented. Times were differentiated between weekdays and weekends, as well as between day shift admissions (7:00–16:00 on weekdays and 9:00–18:00 on weekends) and on-call staff admissions.

Positive ethics approval was obtained from the University of Bonn ethics committee before conducting this study (323-23-EP). For this retrospective study, the ethics committee waived the need for informed consent. Only medical personnel already involved in the treatment and care of patients had access to patient data. The study was conducted in accordance with the principles of the Declaration of Helsinki.

### Statistical analysis

The data were compiled using Microsoft Excel 2024 (Microsoft Corporation, Richmond, Virginia, USA). SPSS Statistics version 28 for Windows (SPSS, Inc., a subsidiary of IBM, Chicago, IL, USA) was used for the statistical analysis. Descriptive statistics were calculated, including arithmetic means, standard deviations and ranges. Numerical variables were expressed as mean (M) ± standard deviation (SD). An alpha error of 5% was considered statistically significant. The chi-squared test with Cramer’s V was used to test for associations between nominal variables. Spearman’s rank correlation coefficient was used to analyse correlating variables. For normally distributed continuous variables (tested using the Shapiro-Wilk test), p values were calculated using an unpaired two-tailed t-test. For non-normally distributed data, the Mann-Whitney U test was employed. To obtain meaningful results, the odds ratio (confidence interval, CI) was analysed to quantify the strength of the association between two variables.

## Results

### Complete collective

A total of 68 patients who had intentionally fallen from a great height (34 male, 34 female) were included in the final analysis. These patients were then compared to a group of 68 patients with unintentional falls from great heights, either work-related or during recreational activities. The unintentional group consisted of 52 male and 16 female patients, with a significantly higher male-to-female ratio (*p* = 0.002). On average, the suicidal group were 42.12 years old (± 17.75), aged between 15 and 88 years. In contrast, the unintentional group had an average age of 42.07 years (± 20.53), spanning from 5 to 94 years of age. Furthermore, the suicidal group jumped from an average height of 9.61 m (± 6.42) compared to an average height of 6.14 m (± 4.08) in the unintentional group (*p* < 0.001). Demographic data for both groups is provided in Table 1.

**Table 1:** Comparison of the demographic data from the suicidal and unintentional group with significant p values highlighted

	Unintentional	Suicidal	*p* value
Age (years)	42.07 (± 20.53)	42.12 (± 17.75)	> 0.05
ASA-Classification	1.84 (± 0.77)	2,13 (± 0.62)	**0.007**
Male	52	34	**0.002**
Female	14	34	
Fall height (meters)	6.14 (± 4.08)	9.61 (± 6.42)	**< 0.001**
Injury Severity Score	17.37 (± 14.01)	32.04 (± 23.43)	**< 0.001**
Intoxication during trauma	17.6%	19.1%	> 0.05

ASA-Classification = American Society of Anesthesiologists Classification

**Fig. 1:**
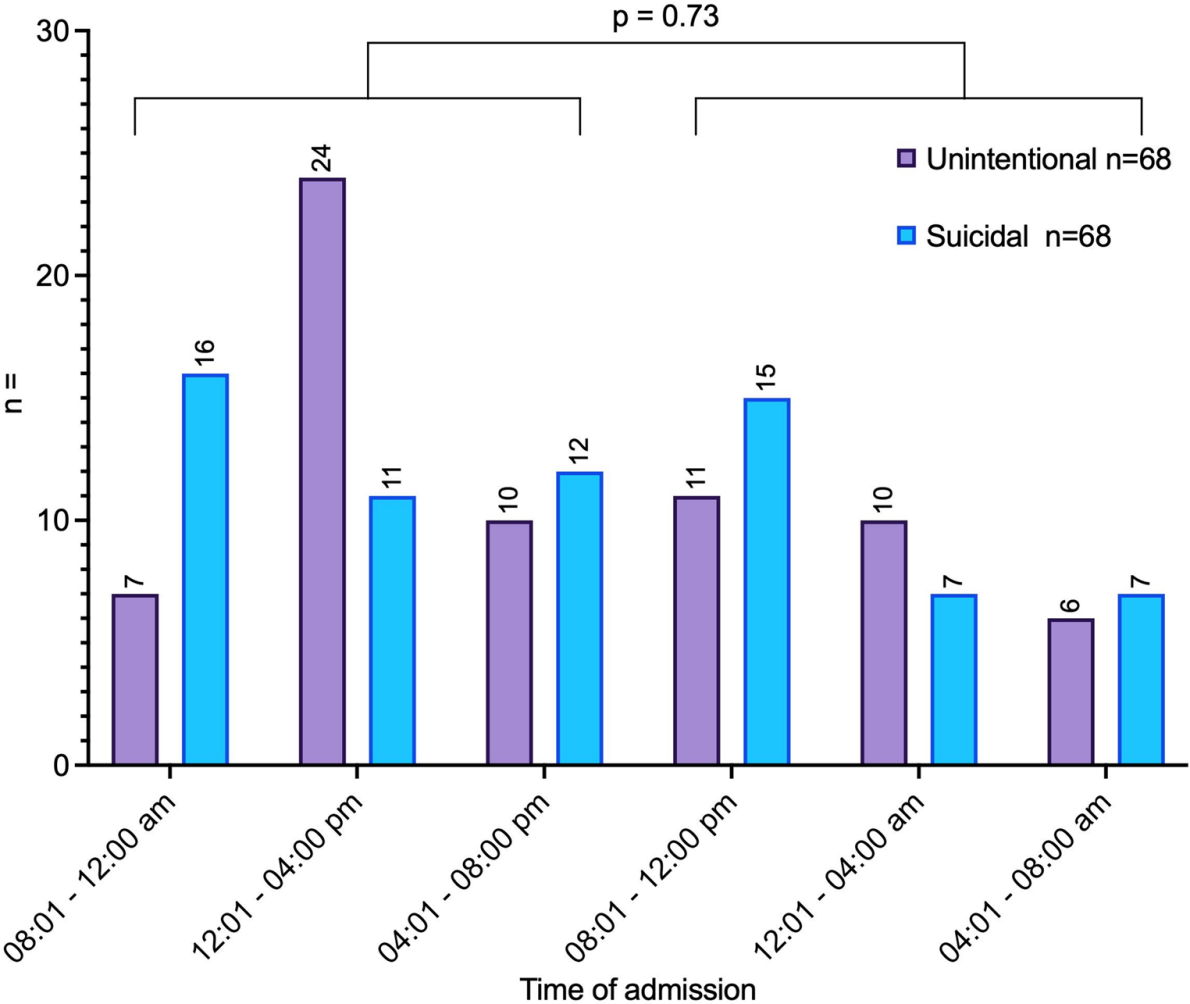
Admission time to the emergency room of the patients following suicidal or unintentional fall trauma

Patients’ arrival times are shown in Fig. 1. There were no differences between the two groups in terms of shift vs. normal working hours (*p* = 0.730) or weekend vs. weekday admission (*p* = 0.475).

Upon arrival at the emergency room, the mean Glasgow Coma Scale (GCS) for the suicidal group was 9.19 (± 5.43), whereas for the unintentional group it was 11.87 (± 4.79) (*p* = 0.001). The mean Injury Severity Score (ISS) for the suicidal group was 32.04 (± 23.43), while the mean ISS for the unintentional group was 17.37 (± 14.01). The suicidal group had a significantly higher ISS (*p* < 0.001). Additionally, the suicidal group experienced significantly more trauma to the lower extremities (*p* < 0.001), the pelvis (*p* < 0.001) and the thorax (*p* = 0.013). The exact injury pattern is shown in Table 2. A comprehensive analysis of the available dataset revealed that the suicidal group exhibited a significantly elevated risk of various types of injury, as indicated by the following odds ratios: 3.34 (95% CI: 1.68–6.98) for pelvic injuries; 2.62 (95% CI: 1.28–5.37) for thoracic injuries; and 3.60 (95% CI: 1.77–7.30) for lower extremity injuries.

**Table 2:** Comparison of the injury patterns of the suicidal group and unintentional group with significant p values highlighted

Injury to the	Unintentional (in %)	Suicidal (in %)	*p* value
Upper extremity	33.8	48.5	*p* > 0.05
Lower extremity	32.4	63.2	***p < 0.001***
Pelvis	29.4	58.8	***p < 0.001***
Thorax	51.5	73.5	***p = 0.01***
Abdomen	23.5	38.2	*p* > 0.05
Outer	97.1	97.1	*p* > 0.05
Face	32.4	35.3	*p* > 0.05
Head/Neck	48.5	50	*p* > 0.05
Spine	54.4	57.4	*p* > 0.05

The lethality of the traumatic injuries in the suicidal group was 25%, which was significantly higher than in the unintentional fall group (10.3%; *p* = 0.041). Fall height was more closely correlated with ISS in the suicide group than in the unintentional fall group (see Table 3). The suicide group was 2.91 times more likely to die in hospital than the unintentional group (confidence interval 1.12–7.55).

**Table 3:** The Spearman-correlation between fall height und ISS score in the two subgroups with a visibly higher correlation between the two parameters in the suicidal group

Subgroups	Injury Severity Score	Fall height
unintentional (N = 68)	ρ = 0.328	ρ = 0.328
	p-value = 0.006 (**)	p-value = 0.006 (**)
suicidal (N= 68)	ρ = 0.405	ρ = 0.405
	p-value < 0.001 (***)	p-value < 0.001 (***)

ρ = Spearman-correlation coefficient.

**Fig. 2:**
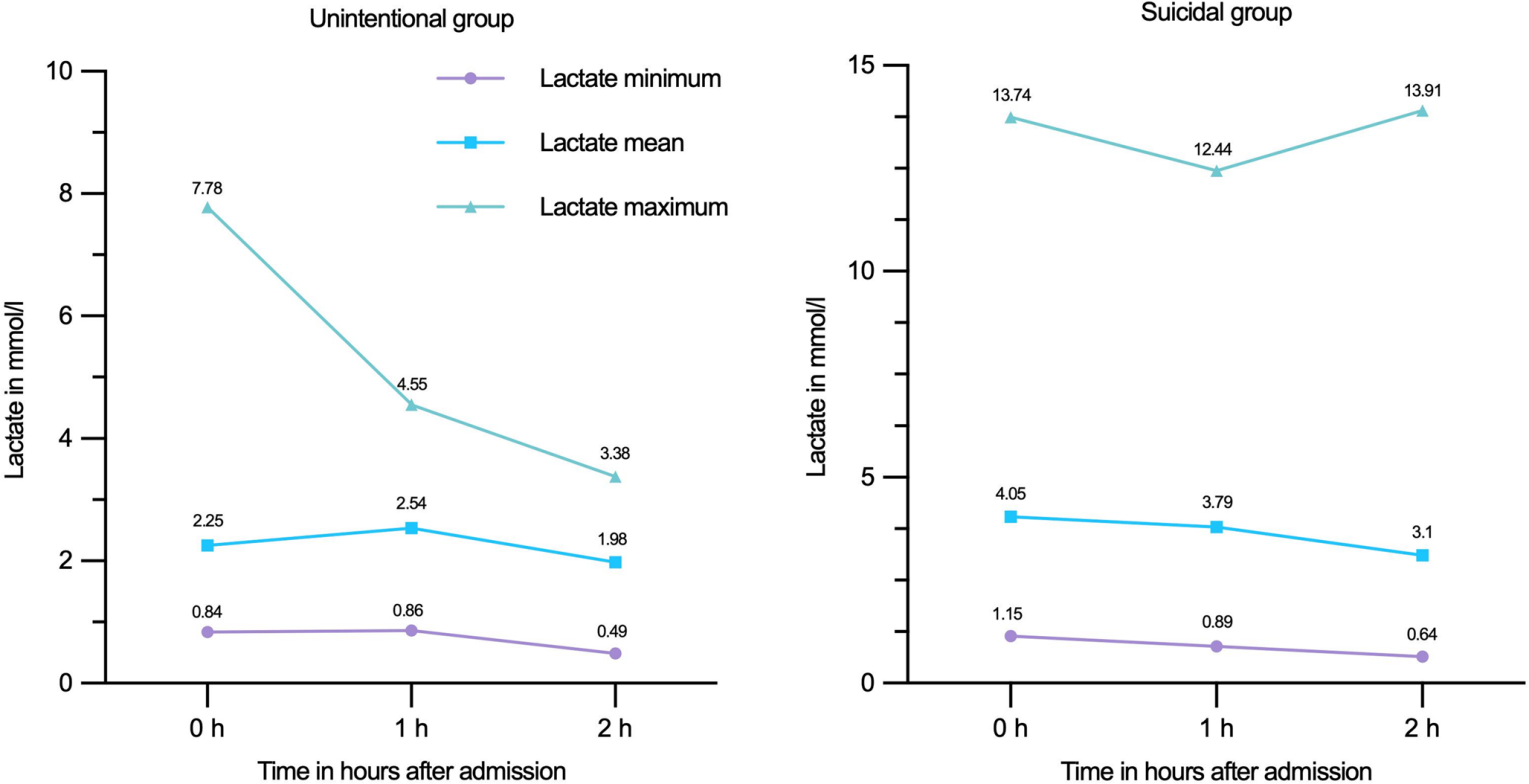
**A** & **B** Suicidal and unintentional group: blood gas analysis for lactate at admission and the following 1 h and 2 h after admission to the emergency room

The initial levels and evolution of arterial blood gas analysis with lactate, base excess and pH levels during the first hours after trauma are presented in Table 4, with significant differences in lactate on arrival to the emergency room. Figure 2A and B illustrate how blood gas analysis parameters changed for the suicide and accident groups.

**Table 4:** A comparison of the pH-value, base excess (BE) and lactate from blood gas analysis between the two groups at the arrival at emergency room (0 h). The values 1 h and 2 h represent the values recorded one and two hours after arrival at the emergency room

Measurement	Unintentional	*N*	Suicidal	*N*	*p* value
0 h pH	7.34 (± 0.08)	65	7.30 (± 0.16)	62	0.677
0 h BE mmol/l	−1.79 (± 3.61)	65	−4.11 (± 6.34)	62	0.059
0 h Lactate mmol/l	2.25 (± 1.29)	65	4.05 (± 2.69)	62	**< 0.001**
1 h pH	7.31 (± 0.07)	23	7.28 (± 0.16)	31	0.993
1 h BE mmol/l	−2.95 (± 3.19)	23	−4.46 (± 6.36)	31	0.820
1 h Lactate mmol/l	2.54 (± 1.11)	23	3.79 (± 3.05)	31	0.178
2 h pH	7.31 (± 0.06)	23	7.32 (± 0.11)	35	0.212
2 h BE mmol/l	−4.02 (± 3.27)	23	−3.06 (± 4.92)	35	0.137
2 h Lactate mmol/l	1.98 (± 0.89)	23	3.10 (± 3.25)	35	0.546

BE: base excess.

Similarly, blood tests were routinely performed upon arrival at the emergency department and the intensive care unit (ICU), and daily thereafter, to analyse white blood cell count, C-reactive protein (CRP), procalcitonin (PCT) and coagulation status following trauma. Table 5 shows the results for the inflammatory markers, with the significant differences between the two groups.

**Table 5:** Inflammatory markers following on day 0 and the first two days following trauma for in the unintentional and suicidal group

Measurement	Unintentional	*N*	Suicidal	*N*	*p*
CRP day 0 (mg/l)	4.22 (± 6.59)	17	37.48 (± 75.45)	13	0.123
CRP day 1 (mg/l)	22.05 (± 16.68)	11	51.18 (± 31.69)	9	**0.038**
CRP day 2 (mg/l)	102.79 (± 87.45)	10	145.55 (± 103.22)	6	0.313
PCT day 0 (µg/l)	1.05 (± 1.43)	16	1.31 (± 2.33)	18	0.695
PCT day 1 (µg/l)	1.15 (± 1.30)	23	5.73 (± 15.77)	17	0.645
PCT day 2 (µg/l)	1.61 (± 3.48)	19	3.58 (± 10.28)	24	0.660
Leukocytes day 0 (G/l)	14.51 (± 8.19)	66	16.88 (± 9.37)	64	0.073
Leukocytes day 1 (G/l)	11.33 (± 6.67)	35	9.88 (± 4.73)	43	0.684
Leukocytes day 2 (G/l)	10.38 (± 3.10)	32	9.72 (± 3.81)	44	0.192

CRP: c-reactive protein; PCT: procalcitonin

The coagulation status, alongside its significant differences, is reported in Table 6. Body temperature, which can affect coagulation status, was also measured upon arrival at the ER and was, on average, 35.87 °C (± 1.05) in the unintentional group and 35.85 °C (± 1.36) in the suicidal group, with no significant difference between the two.

**Table 6:** Comparison of the coagulation status and blood transfusions during trauma management between the two groups

Measurement	Unintentional	*N*	Suicidal	*N*	*p* value
D-Dimers in mg/l	22.33 (± 13.84)	28	27.48 (± 10.64)	35	0.078
prothrombin time in %	89.09 (± 21.66)	66	74.95 (± 21.89)	60	** < 0.001**
Fibrinogen in mg/dl	219.93 (± 87.58)	28	208.35 (± 107.66)	34	0.320
Erythrocyte concentrates	0.97 (± 2.29)	68	4.07 (± 9.39)	68	** < 0.001**
Thrombocyte concentrates	0.18 (± 0.55)	68	0.5 (± 1.69)	68	0.373
Fresh-Frozen-Plasma	0.84 (± 2.29)	68	2.74 (± 6.81)	68	** 0.023**

The ICU also tests for vitamin D, total protein and albumin to assess the patient’s nutritional status. No significant differences were detected in vitamin D (*p* = 0.740), total protein (*p* = 0.423) or albumin (*p* = 0.730) levels between the two groups.

Most of the treated patients underwent surgery. On average, the suicidal group received 3.21 (± 4.27) operations per patient, which was significantly more than the unintentional group (1.69 ± 2.81) (*p* = 0.016). There were significant differences in complication rates between the two groups, with the suicidal group experiencing more wound healing disorders (*p* < 0.001) and complications in general (*p* = 0.011). However, there were no significant differences in infections, acute respiratory distress syndrome, neurological deficits, or surgical revision rates.

Following emergency treatment, 72.1% of patients were transferred to the ICU, significantly higher than the 48.5% of patients in the unintentional group (*p* = 0.008). However, a potential bias arises here because acutely suicidal patients required monitoring for their risk of suicide, as well as for medical reasons. The unintentional group was significantly more likely (*p* = 0.022) to be admitted to an intermediate care unit (38.2%), compared with 19.1% in the suicidal group. The total cumulative ICU and IMC stay was not significantly longer for the suicidal group, at 15.69 days (± 21.1), compared to 8.63 days (± 20.39) for the unintentional group (p = 0.052).

### Subgroup analysis

Due to the significant difference in fall heights (9.61 m (± 6.42) vs. 6.14 m (± 4.08), *p* < 0.001), which introduces an inherent bias to the total collective, we conducted a subgroup analysis by adjusting the inclusion parameters regarding fall height. In this analysis, we only included patients who had fallen from a height of up to 25 m, and we adjusted the minimum fall height of the unintentional group to five meters. This reduced the size of the suicidal group to *n* = 65 and the unintentional group to *n* = 37, but produced a more homogeneous collective regarding fall height, thereby increasing the validity of the comparative analysis. The demographic data for these two groups with the adjusted inclusion parameters are presented in Table 7.

**Table 7:** Comparison of the demographic data from the subgroup analysis of the suicidal and unintentional group with significant p values highlighted

Measurement	Unintentional	Suicidal	*p* value
Age (years)	35.35 ± 18.27	42.69 ± 17.66	**0.049**
ASA-Score	1.68 ± 0.63	2.14 ± 0.63	**0.001**
Male	29	31	**0.002**
Female	8	34	
Fall height (meters)	8.41 ± 4.38	8.52 ± 3.80	0.494
ISS	19.92 ± 16.32	31.18 ± 22.41	**0.012**
GCS	11.19 ± 5.27	9.29 ± 5.39	0.052

ASA-Classification = American Society of Anaesthesiologists Classification; ISS = Injury severity score; GCS = Glasgow coma scale.

As demonstrated, the patients in the suicidal group were significantly older (42.69 ± 17.66 vs. 35.35 ± 18.27, *p* = 0.049) and had significantly higher ASA (2.14 ± 0.63 vs. 1.68 ± 0.63, *p* = 0.001) and IS (31.18 ± 22.41 vs. 19.92 ± 16.32, *p* = 0.012) scores. The unintentional group had a significantly higher proportion of male patients (*p* = 0.002). There were no significant differences in arrival time to the ER between the two groups for shift vs. normal working hours (*p* = 0.690) or admission on the weekend vs. weekday (*p* = 0.636).

The exact injury pattern of the subgroup analysis is shown in Table 8. A comprehensive analysis of the available dataset revealed that the suicidal group exhibited a significantly higher risk of pelvic (OR: 2.60, 95% CI: 1.13–6.00.13.00) and thoracic (OR: 2.40, 95% CI: 1.03–5.62) injuries compared to the unintentional group, as indicated by the odds ratios. Table 9 shows the initial levels and evolution of arterial blood gas analysis with lactate, base excess and pH levels during the first hours after trauma. There were significant differences in lactate levels (mmol/l) on arrival at the emergency room (4.10 ± 2.72 vs. 2.37 ± 1.43, *p* < 0.001). Unlike the full group of 68 patients, no significant differences in post-traumatic CRP, PCT or leucocytes were found in the subgroup analysis.

**Table 8:** Comparison of the injury patterns of the suicidal subgroup and unintentional subgroup with significant p values highlighted

Injury to the	Unintentional (in %)	Suicidal (in %)	*p* value
Upper extremity	32.4	47.7	0.133
Lower extremity	45.9	63.1	0.093
Pelvis	35.1	58.5	**0.023**
Thorax	54.1	73.8	**0.041**
Abdomen	32.4	36.9	0.648
Outer	100	96.9	0.281
Face	37.8	33.8	0.685
Head/Neck	45.9	49.2	0.750
Spine	51.4	55.4	0.694

**Table 9:** A comparison of the pH-value, base excess (BE) and lactate from blood gas analysis between the two subgroups at the arrival at emergency room (0 h). The values 1 h and 2 h represent the values recorded one and two hours after arrival at the emergency room

Measurement	Unintentional	*N*	Suicidal	*N*	*p* value
0 h pH	7.32 ± 0.09	35	7.30 ± 0.16	61	0.741
0 h BE (mmol/l)	−2.33 ± 4.06	35	−4.21 ± 6.35	61	0.204
0 h Lactate (mmol/l)	2.37 ± 1.43	35	4.10 ± 2.72	61	**< 0.001**
1 h pH	7.32 ± 0.08	16	7.28 ± 0.16	31	0.670
1 h BE (mmol/l)	−2.31 ± 3.24	16	−4.46 ± 6.36	31	0.357
1 h Lactate (mmol/l)	2.42 ± 1.09	16	3.80 ± 3.05	31	0.144
2 h pH	7.33 ± 0.06	15	7.32 ± 0.11	35	0.743
2 h BE (mmol/l)	−2.58 ± 2.90	15	−3.06 ± 4.92	35	0.924
2 h Lactate (mmol/l)	1.92 ± 0.94	15	3.10 ± 3.25	35	0.409
BE: base excess					

The coagulation status, which shows significant differences in the subgroup analysis, is reported in Table 10. Body temperature, which can affect coagulation, showed no significant difference between the two groups. However, significant differences were found in prothrombin time (%): 75.97 ± 20.68 vs. 86.03 ± 23.15 (*p* = 0.028), and in the number of erythrocyte concentrates administered to patients: 4.17 ± 9.57 vs. 1.05 ± 2.53 (*p* = 0.002).

**Table 10:** Comparison of the coagulation status, transfusions, and temperature during trauma management between the two subgroups

Measurement	Unintentional	*N*	Suicidal	*N*	*p* value
D-Dimers mg/l	21.63 ± 13.82	16	27.62 ± 10.72	34	0.077
Prothrombin time in %	86.03 ± 23.15	36	75.97 ± 20.68	58	**0.028**
Fibrinogen md/dl	223.88 ± 114.43	16	208.35 ± 107.66	34	0.581
Erythrocyte concentrate	1.05 ± 2.53	37	4.17 ± 9.57	65	**0.002**
Thrombocyte concentrates	0.14 ± 0.48	37	0.52 ± 1.73	65	0.256
Fresh frozen plasma	0.97 ± 2.73	37	2.80 ± 6.95	65	0.074
Temperature	35.99 ± 1.12	28	35.87 ± 1.39	49	0.497

The in-house mortality of traumas in the suicidal subgroup was 23.1%, which was significantly higher than in the unintentional groups (2.7%; *p* = 0.007). Compared to the unintentional groups, the suicidal subgroup exhibited an odds ratio of 10.8 for in-hospital mortality (95% confidence interval: 1.36–85.51).

There were significant differences in the complication rate between the two groups, with the suicidal group having more wound healing disorders (54.5% vs. 38.9%; *p* = 0.021). However, there were no significant differences in general complications, infections, acute respiratory distress syndrome, neurological deficits or surgical revision rates.

## Discussion

### Cohort size and demographic data

Our findings are consistent with existing literature that compares trauma outcomes in cases of unintentional versus suicidal falls. Previous studies examining suicidal falls have included cohorts of between 40 and 149 patients who were alive upon admission to hospital [23, 34, 35]. Larger studies featuring a high number of cases of suicidal falls have typically been conducted over extended periods or have been based on post-mortem analyses. The demographic characteristics reported in the literature are consistent with our data, with most patients reported to be aged between 40 and 45 years old. Furthermore, comparative studies have indicated a significantly higher proportion of males in the unintentional fall group. This trend is also reflected in our dataset. The reported mean fall height for suicidal cases ranges from 7.9 to 10.4 m. Our average fall heights of 9.61 and 8.41 m, as determined by subgroup analysis, are therefore in line with the available literature [23, 35].

### Trauma severity

The average ISS was 32.04 (± 23.43) for the suicidal group and 17.37 (± 14.01) for the unintentional group. The suicidal group experienced significantly more trauma to the lower extremities, pelvis and thorax, which largely accounts for the significantly higher in-hospital mortality observed in this group. Subgroup analysis, in which fall height did not differ significantly, showed a similar trend, with a higher ISS and significantly more trauma to the pelvis and thorax. This highlights the severity of the trauma associated with suicide attempts involving jumping. At present, we can only speculate as to why falls from similar heights can result in such differences in ISS and injury patterns. Biomechanically, it is suspected that accidental falls involve more reflexive movements and land more asymmetrically, which may lengthen the stopping distance, help to shed energy, and protect vital organs. Intoxication with alcohol or other substances can affect reflexive movements during a fall and worsen coagulation status. However, there were no significant differences between the cohorts (17.6% vs. 19.1%; *p* > 0.05), so intoxication can be ruled out as a contributing factor. Furthermore, scene selection (e.g. falling on grass vs. concrete/asphalt) and protective gear worn by the patient in work-related falls can help explain the differences observed in these cohorts. While it is possible to suspect, it remains difficult to prove, that a person intending to commit suicide may select a more harmful landing surface in order to maximise injury. Exploring this preclinical data in relation to these traumas is difficult and lacking in the current literature on this topic.

Similarly to our cohort, Kort et al. (2023) found that the suicidal group fell from a greater height than the unintentional group (10.4 ± 7.3 m vs. 7.1 ± 5.7 m), and reported more injuries to the abdomen, pelvis and upper and lower extremities [35]. Pelvis injuries were 2.1 times more likely to occur after a suicidal fall than an unintentional one, which is slightly below the data for our cohort [35]. In terms of injury severity, this study’s cohort is among the most severely injured reported in the literature. Mortality was also significantly higher within the suicidal group, at 25%, compared to 10.3% in the unintentional group. Even when fall height was adjusted for, the mortality rate in the suicidal group was still significantly higher, at 23.1%, compared to 2.7% in the unintentional group. Piazzalunga et al. also report significantly higher mortality for the suicidal group (7.5% vs. 1.2%) [23]. The higher mortality rates seen in our cohort can be explained by the lower Injury Severity Score (ISS) (18.88 ± 11.80) and fall height (7.85 ± 5.82) observed in Piazzalunga et al.‘s study. Our data and the existing literature indicate that trauma resulting from falls intended as a means of suicide is generally more severe, even when adjusted for fall height. This is associated with a higher ISS, in-hospital mortality, and pelvic and thoracic injuries, which can significantly impact clinical outcomes compared to unintentional falls.

### Inflammatory response

To analyse the inflammatory response of patients to trauma following falls from height, BGA and inflammatory parameters were analysed in the first three days after trauma. This aspect of trauma management for these patients is not yet present in the available literature. As is well known, fall height correlates with the ISS of patients, a finding that was also consistent in our dataset and demonstrated above [36, 37]. In cases of suicidal falls, the accuracy of the patient’s account of the height of the fall is often compromised, resulting in an inaccurate and incomplete record. The objective of this study was therefore to determine whether there was a correlation between the patients’ inflammatory response and the severity of their trauma. A statistically significant difference in lactate levels was observed between the two groups. The suicidal group exhibited an average lactate level of 4.05 (± 2.69) mmol/L, while the unintentional group demonstrated an average level of 2.25 (± 1.29) mmol/L. This remained true in the subgroup analysis, where the suicidal group exhibited an average lactate level of 4.10 (± 2.72) mmol/L and the unintentional group an average of 2.37 (± 1.43) mmol/L (*p* < 0.001). A strong correlation was observed between 0-, 1- and 2-hour lactate levels and ISS within the suicidal group. In the unintentional group, this correlation was only strong upon admission, weakening at hours one and two after admission. This is consistent with the dataset of Cerović et al. (2003), who prospectively analysed 98 severely injured patients, 25.5% of whom died [38]. In our case, as in the aforementioned study, regression analysis was able to predict injury severity (ISS) based on admission lactate. Another study by Okello et al. (2014), which analysed 108 severely injured patients, demonstrated that an admission lactate level of >2 mmol/L could discriminate between severe and non-severe injuries, with 88% sensitivity and 38% specificity [39].

Regarding PCT levels in patients following trauma, a positive correlation with the ISS was observed, with higher values on average in the suicidal group, though no significant differences were found between the groups. Various studies have been conducted on this topic, including those by Hoshino et al., Maier et al., and Wojtaszek et al. [28–30]. These studies have consistently shown a positive correlation between PCT levels and severe injury. In fact, some studies have even identified PCT as a prognostic marker for trauma-related complications [28–30]. A systematic review by AlRawahi et al. (2023), which included 19 studies of severely injured patients, confirmed the role of PCT as a marker of severe trauma and of complications [40].

However, it should be noted that psychiatric medications, particularly selective serotonin reuptake inhibitors (SSRIs), are thought to influence immune function. There is evidence to suggest that they reduce pro-inflammatory cytokines. Nevertheless, the relationship between depression and inflammation remains complex and has not yet been definitively established [41]. This may have acted as a confounding factor when analysing a cohort with psychiatric comorbidities.

### Complications

This study revealed a significantly higher prevalence of wound healing disorders requiring revision surgery in the suicidal group than in the unintentional group. This finding was consistent in both the general and subgroup analyses.

The impaired wound healing experienced by patients who have attempted suicide by jumping may be influenced by various psychological and local monocentric interdisciplinary factors. A comprehensive review and meta-analysis conducted by Walburn et al. (2009) supports an association between psychological distress and impaired wound healing [42]. Additionally, evidence suggests a correlation between depression and impaired wound healing. This was observed in patients recovering from heart surgery in a study conducted by Doering et al. (2005) [43]. In our local setting, poorer outcomes may have been due to challenges in coordination and management between the psychiatric ward and the trauma centre, which were involved in interdisciplinary treatment. Furthermore, patients who have attempted suicide may be less compliant than those who have experienced unintentional trauma. This further complicates the healing process. As demonstrated in the study by Dew et al., mental health status has been shown to significantly impact postoperative physical morbidity and mortality in patients undergoing heart surgery [44].

Although hypoalbuminemia and low vitamin D status are recognised risk factors for postoperative wound complications, they cannot account for the excess observed in the suicidal group, as cohort albumin (*p* = 0.730) and vitamin D (*p* = 0.740) levels were comparable [45–47].

### Time of injury

The study’s initial hypothesis proposed that suicidal injuries would be more prevalent during hospital late- and night shifts and on weekends than unintentional falls. Given the established correlation between the time of arrival at hospital and the subsequent risk of complications and mortality, we considered the time of arrival to be a potential confounding factor in our analysis [48, 49]. Nevertheless, the findings of this study enabled this parameter to be excluded as a confounding factor in the observed differences in outcomes between the two groups, as well as in the subgroup analysis. These results support the hypothesis that other variables may play a more significant role in group-specific complication and mortality rates.

### Coagulation status

The data from this study indicate that trauma patients presenting after suicidal falls exhibit significantly worse coagulation status. This is evident from the poorer prothrombin times observed upon arrival in the ER, as demonstrated in both the general and subgroup analyses. This impaired coagulation profile is clinically relevant as it is associated with an increased risk of bleeding complications and the need for higher volumes of blood and fresh frozen plasma transfusions. Although hypothermia is a recognised cause of coagulopathy in trauma patients, it was eliminated as a confounding factor in our analysis as there were no significant differences in admission temperatures between the two groups. These differences in coagulation status may be explained by more severe sustained injuries with a significantly higher ISS, as prothrombin time is negatively affected by this and can even serve as a predictive value in polytrauma patients [50].

Another contributor to the impaired coagulation status observed in the patients upon arrival at the emergency room may have been psychiatric medication taken by some patients with suicidal intent. Selective serotonin reuptake inhibitors block the serotonin transporter on platelets, depleting intraplatelet serotonin by approximately 80–90% over time and thereby affecting primary haemostasis [51]. Consistent with this mechanism, Auerbach et al. (2013) reported that surgical patients receiving SSRIs experienced higher in-hospital mortality, more perioperative bleeding, and increased 30-day readmissions, which could partly explain the differences in the cohorts observed in this study [52]. Taken together, these data support incorporating SSRI exposure into early haemostatic risk stratification and perioperative planning in this cohort.

In summary, these patients should be monitored closely for haemorrhagic complications.

### Limitations

The first limitation of this study was its retrospective design and the lack of clinical follow-up. This could have resulted in critical complications in trauma surgery being overlooked, such as non-unions, mechanical irritation of plates/screws, late hardware infections, lasting numbness over scars and secondary arthritis following trauma. Furthermore, long-term follow-up could provide valuable information regarding functional outcomes following complex surgical treatment. Additionally, estimation of fall height must be regarded as a potential limitation of the results, given that unintentional falls, especially in a work-related setting, could be more accurately estimated due to the presence of witnesses and the more controlled environment. Suicidal falls often occur without witnesses, meaning that the fall height must be estimated by first responders. Psychiatric illness leading to suicidal falls may have further contributed to complications and worse long-term outcomes in the suicidal cohort.

Furthermore, in some cases, the height and mechanism of the fall had to be estimated as there were no eyewitnesses at the injury site. Additionally, pre-hospital trauma management and mortality data were unavailable for both groups, which may have skewed the results.

## Conclusion

This retrospective, single-centre comparative analysis of patients who had fallen from a height found that suicidal falls were associated with substantially more severe injury patterns and worse early outcomes than unintentional falls. The mean ISS was almost double in the suicidal group (32.04 ± 23.43 vs. 17.37 ± 14.01; *p* < 0.001) and remained so after adjusting for fall height (31.18 ± 22.41 vs. 19.92 ± 16.32; *p* = 0.012). In-hospital mortality was over twice as high among suicidal patients (25.0% vs. 10.3%; *p* = 0.041), with an even greater difference observed in the height-adjusted cohort (23.1% vs. 2.7%; *p* = 0.007). Suicidal patients also required more transfusions and operations, and experienced higher rates of wound-healing complications (*p* = 0.021). Overall, suicidal falls represent a distinct type of high-energy trauma, characterised by greater physiological derangement upon arrival and higher early resource consumption. This was highlighted by lactate levels (mmol/L) upon admission, which were significantly higher in the suicidal group (4.10 ± 2.72) than in the unintentional group (2.37 ± 1.43) (*p* < 0.001). Furthermore, the data showed a tendency towards a greater inflammatory response in the suicidal group, with significantly higher C-reactive protein levels on day 1 following trauma (*p* = 0.038) and procalcitonin levels after the first two days following trauma. These findings remained consistent after accounting for fall height.

In summary, special attention should be paid to the frequent occurrence of complications, increased transfusion requirements, and significantly impaired coagulation status in these patients upon hospital admission. Confounding factors such as hypothermia and time of admission could not be identified in this study, but should always be considered when treating patients with high-energy trauma, such as those who have fallen in an attempt to take their own life.

## Data Availability

The data that support the findings of this study are not openly available due to reasons of sensitivity and german privacy laws. Data may be available from the corresponding author upon reasonable request and if german privacy laws do not prohibit sharing of these data. Data are located in controlled access data storage at the University of Bonn.

## Abbreviations

ISS: Injury severity score
ASA: American Society of Anaesthesiologists
ER: Emergency room
GCS: Glasgow coma scale
ICU: Intensive care unit
IMC: Intermediate care unit
CRP: C-reactive protein
PCT: Procalcitonin
BE: Base excess
BGA: Blood gas analysis
